# Different Pathways Leading up to the Same Futsal Competition: Individual and Inter-Team Variability in Loading Patterns and Preseason Training Adaptations

**DOI:** 10.3390/sports7010007

**Published:** 2018-12-28

**Authors:** Anderson Santiago Teixeira, Renan Felipe Hartmann Nunes, Javier Yanci, Pascal Izzicupo, Lucinar Jupir Forner Flores, João Carlos Romano, Luiz Guilherme Antonacci Guglielmo, Fabio Yuzo Nakamura

**Affiliations:** 1 Physical Effort Laboratory, Sports Center, Federal University of Santa Catarina, Florianópolis–SC 88040-900, Brazil; nunesrenan85@hotmail.com (R.F.H.N.); luiz.guilherme@ufsc.br (L.G.A.G.); 2 Faculty of Education and Sport, University of the Basque Country, UPV/EHU, E-01006 Vitoria-Gasteiz, Spain; javier.yanci@ehu.eus; 3 Department of Medicine and Aging Sciences, University Gabriele d’Annunzio of Chieti-Pescara, 66100 Chieti-Pescara, Italy; pascalizzicupo@gmail.com; 4 Department of Physical Education, State University of Western Parana, Marechal Candido Rondon, Paraná 85960-000, Brazil; lucinar05@gmail.com; 5 Joinville Esporte Clube/Krona Futsal, Joinville 89202-310, Brazil; joaocarlosromano@gmail.com; 6 Confederação Brasileira de Futsal, Fortaleza 60060-150, Brazil; 7 The College of Healthcare Sciences, James Cook University, Queensland 4811, Australia; 8 Department of Physical Education, Federal University of Paraiba–UFPB, João Pessoa–PB 58051-900, Brazil

**Keywords:** indoor soccer, team sports, training adaption, vertical jump, sprint performance

## Abstract

During the preseason, futsal players deal with large internal load, which may result in a reduction in physical performance. The aims of this study were to compare the session rating of perceived exertion training load (s-RPE TL) during the preseason between two teams; and to analyze the changes on the delayed-onset muscle soreness (DOMS), aerobic- and speed-power characteristics in players accumulating different s-RPE TL (Low (LTL) vs. High (HTL)). Twenty-eight players (Team A, n = 15; Team B, n = 13) were recruited. The s-RPE TL was monitored throughout the preseason phase (five weeks) in both teams. The coaches of each team planned the activities that comprised their training programs, without any interference from the researchers. Team A evaluated countermovement jumps (CMJ) and DOMS weekly. Team B performed squat jumps (SJ), CMJ, 5 m and 15 m sprints, and a futsal intermittent endurance test (PV_FIET_) before and after the preseason. Team B accumulated an almost-certainly greater s-RPE TL than Team A. In Team A, the CMJ height was likely to almost certainly improved for the HTL group from week 3. In Team B, the 5 m and 15 m sprint likely decreased after the preseason. Changes in 5 m (r = −0.61) and 15 m (r = −0.56) were correlated with total s-RPE TL. Changes in PV_FIET_ were positively associated with changes in sprint, but inversely related to the baseline. s-RPE TL differed between both teams, and substantial gains in neuromuscular performance were observed for the HTL group in Team A. Slower and faster players in Team B showed distinct intermittent-endurance and speed adaptive responses during the high-volume preseason.

## 1. Introduction

Futsal is an intermittent team sport characterized by high-intensity efforts and frequent multidirectional sprinting activities during official matches [1], requiring players with a high level of athletic performance in a multitude of physical abilities. Technical–tactical activities can represent up to 50–70% of the total training volume during the preseason phase in a professional futsal team [2]. It means that strength and conditioning specialists have about 30–50% of the remaining training time to optimize a variety of futsal-related physical fitness components, such as strength and power–speed characteristics. Certainly, any training program during the preseason phase is planned, aiming to maximize positive training outcomes (i.e., fitness improvements) and to minimize negative consequences (i.e., injury, fatigue and overtraining) [3]. Thus, to allow players to be physically and mentally prepared to face the sequence of games during the in-season period, it is fundamental that training loads and recovery status (e.g., delayed-onset muscle soreness (DOMS)) are regularly monitored on an individual basis [4].

Currently, the session rating of perceived exertion (s-RPE) is one of the simplest, but still useful, reliable, valid and inexpensive methods that are commonly used to quantify internal training load (ITL), providing a global indicator of exercise intensity in team sports [5,6]. The preseason phase is the period in which the players accumulate the highest s-RPE training load (s-RPE TL) throughout the entire season after returning from a medium to long period without training (off-season) [2,7]. These high pre-season TL are generally associated with higher monotony, strain, poor sleep and greater stress, fatigue, and DOMS than during the in-season period [8]. Thus, the balance between the magnitude of TL applied and the athlete’s recovery state is what will determine whether the training-induced adaptations will be positive or negative. It is well accepted that an athlete’s recovery during the training process is essential for prevent and minimize the incidence of syndromes that impair the athlete’s performance and general health, such as non-functional overreaching or overtraining [9,10,11,12]. For instance, Nakamura et al. [13] showed substantial decreases in vertical jumping ability and sprinting speed after nine weeks of preseason in under-20 years (U-20) professional futsal players. The decreases in jump height and sprint ability reported by Nakamura et al. [13] can be indicative of either maladaptations to the training stimulus caused by the accumulation of fatigue as a result of insufficient recovery time, or an inappropriate or insufficient training stimulus to elicit the desired adaptations. Another possibility would be the concurrent training effects [14] caused by the interference effect between endurance and neuromuscular training [15] extensively performed during this period of preparation.

In addition to the quantification of the TL experienced by the athlete, monitoring changes in physical fitness parameters and DOMS scores is considered to be essential in the training process, especially to identify the athlete’s readiness, and to verify whether they are adapting to the training program [4]. As team sport athletes during the preseason period need to rapidly increase their physical performance, s-RPE TLs are naturally intensified [7]. There are studies that are carried out with both individual [16] and team sports [17,18], supporting the concept that the longer the athlete spends training in high-intensity zones, the greater the gains in crucial components that are related to aerobic running performance. In team sports, probably because of the high volume of technical–tactical training (aerobic predominance), the dose-response relationship with regard to neuromuscular and sprinting abilities does not seem to follow the same pattern of the aforementioned concepts for aerobic running performance. For instance, intensified TL has been linked to decreased neuromuscular ability in soccer [19], rugby [11], and basketball players [20]. Particularly in futsal, this relationship still needs to be better elucidated. Previous studies have showed no relationship between TL that is accumulated, and changes in neuromuscular measures in futsal players [13,21]. A practical approach to understand the relationship between TL and performance is to compare the changes in neuromuscular performance measures between players who accumulate a high (HTL) and low s-RPE TL (LTL) during the preseason phase. If the assumption that an excessive TL accumulated during preparation period impairs neuromuscular ability in team sport athletes is correct, it would be expected that a higher decrement of power–speed performance is found in players with HTL.

Whilst objective and relevant data have been published, regarding the monitoring of s-RPE TLs during the preseason phase in futsal players [2,21,22], most of these previous studies have evaluated a single team in their designs. This can be a limitation, since the reported results may be specific to these players and to the planning that is adopted by the coaching staff of these particular clubs. Therefore, further studies describing the magnitude of s-RPE TL and the distribution of the types of training during the preseason phase involving different professional futsal teams belonging to the same elite level National League are also warranted. The resulting information may be of paramount practical relevance to coaches and strength and conditioning professionals, as it would allow for a better understanding of how futsal athletes train across different teams, and highlight how s-RPE TLs are organized during this very short period of training, leading up to competition.

Therefore, the purposes of the present study were: (1) to describe and compare the typical s-RPE TL accumulated during the preseason phase between two professional Brazilian futsal teams from the First Division League (Team A and B); (2) to compare the weekly changes on neuromuscular performance and DOMS in players with different s-RPE TLs (LTL vs. HTL) accumulated during 5 weeks of preseason in Team A; (3) to analyze the training-induced changes on aerobic performance, neuromuscular ability and sprinting speeds during the 5 weeks of preseason in futsal players, and to explore possible associations with s-RPE TLs and changes in performance in Team B.

## 2. Materials and Methods

### 2.1. Participants

Twenty-eight elite male futsal players (age: 27.0 ± 5.4 year, height: 176.1 ± 3.1 cm, body mass: 73.1 ± 6.8 kg) from two Brazilian professional teams (Team A (n = 15; age: 31.0 ± 5.8 year, height: 177.0 ± 5.2 cm, body mass: 74.4 ± 7.7 kg) and Team B (n = 13; age: 23.0 ± 4.9 year, height: 175.2 ± 1.0 cm, body mass: 71.8 ± 5.9 kg)) volunteered to participate of this study. Players who did not meet the data inclusion process or did not participate in at least 75% of monitored training sessions were excluded. These selected futsal players competed in the First Division of the Brazilian Futsal League; and some of them have played for the Brazilian National senior futsal team (n = 7).

Before commencement of the study, all players were familiar with the study procedures. While approval to conduct the study was granted by the technical staffs of the involved teams, the present data arose as a condition of the monitoring procedures that were regularly performed by the respective teams. Therefore, because of the retrospective nature of the analyses without interfering in the training routine, signatures of the informed consent form were not required [23]; nevertheless, to ensure player confidentiality, all physical performance data were anonymized before analyses.

### 2.2. Design and Procedures

The whole study was conducted during the first five weeks of the preseason phase (2017 season) for both teams. In Team A, players had their countermovement jump (CMJ height) performance measured weekly (each Friday morning), and their perception of DOMS was computed daily before the first daily training session. In Team B, players were assessed for a variety of physical performance measures at baseline (week 0) and after the five weeks of preseason training period: (i) vertical jump tests (CMJ and SJ), (ii) linear 15 m sprint speed, and (iii) a futsal intermittent endurance test (FIET). During the study period, the ITL of each player was also monitored by the means of the s-RPE method [5]. Based on total s-RPE TL accumulated over five weeks of the preseason, players were categorized into two groups (LTL and HTL) by using the median split technique.

### 2.3. Training Program

**Figure 1:**
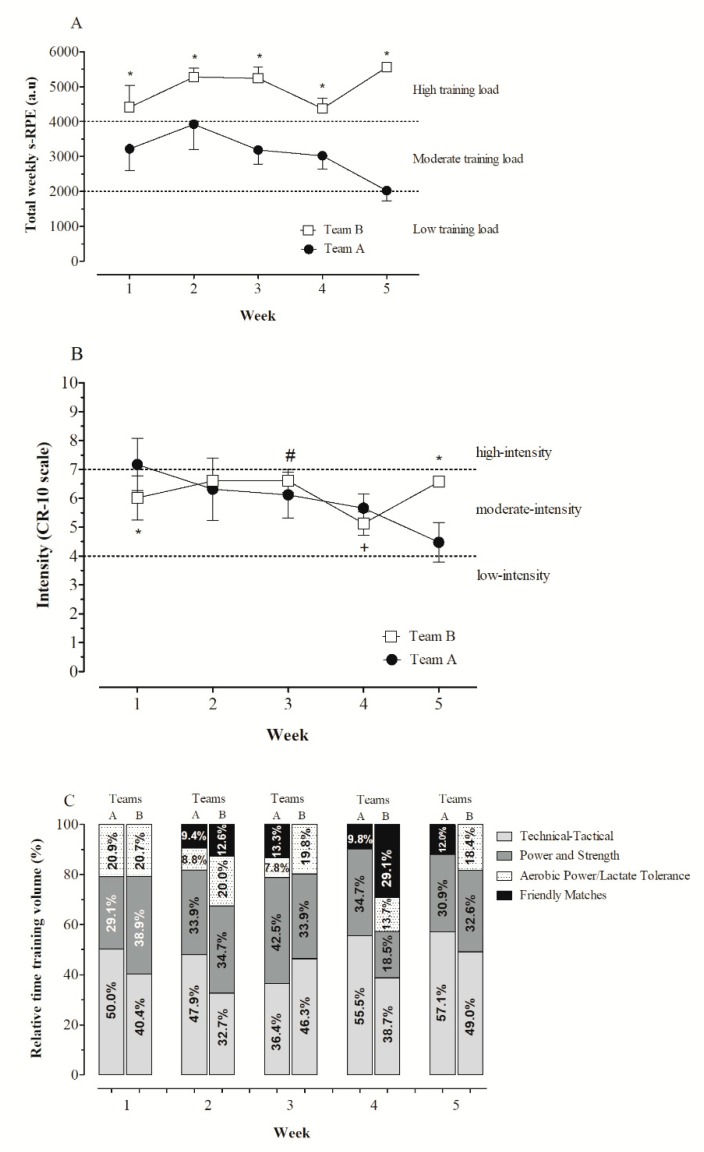
Comparisons of the total weekly s-RPE TL (panel (**A**)) and training intensity (RPE score) (panel (**B**)) between both the teams A and B. Training volume is represented as percentage of time devoted to technical–tactical training sessions, strength and power, aerobic power/lactate tolerance, and friendly matches during the five weeks of the preseason phase for both the teams (panel (**C**)). Threshold values used for training load zones (panel (**A**)) and training intensity zones (panel (**B**)) were established based on the studies of Miloski et al. [2] and Moreira et al. [7], respectively. Note: * *almost certain*; + *very likely*; # *likely* differences between Teams A and B.

The training programme during the preseason phase was planned by technical staff without any intervention or advice provided by the authors. All training sessions (including the baseline week) were regularly monitored. Sessions dedicated to the development of tactical and/or technical ability were referred to as “technical–tactical training”; training sessions devoted to the improvement of neuromuscular ability by using resistance and plyometric exercises were referred to as “strength-power training”; interval training strategies and small-sided games were classified as “aerobic power/lactate tolerance training”; matches against other teams were referred to as “friendly matches”. Briefly, the athletes engaged in a training program of two sessions per day, five days per week. The players woke up at ~6:00–7:00 a.m. to attend a training session starting at 9:00 a.m., lasting for ~90–120 min. After a ~4 h period of recovery (at 3:00 p.m.), players attended the second training session of the day, lasting for ~90–120 min. Specifically, training was aimed at developing technical–tactical abilities, power and strength, aerobic power, and tolerance to lactic acidosis and friendly matches (Figure 1C). During the study period, in order to minimize the impact of confounding factors, the athletes (Team A and B) did not take part in any other training routines (except the training program planned by the teams’ coaching staff). The consumption of water or energy drinks during and post-training was ad libitum.

### 2.4. Neuromuscular Performance Tests

Neuromuscular performance tests were assessed by (1) vertical jump variables and (2) linear 15 m sprint speed. All participants starting with 10–15 min of a standardized warm-up stage that consisted of stretching, jogging, jumps, and sprints with changes of direction [2]. The CMJ (Team A and B) and SJ (Team B) were performed to assess jumping height. In the CMJ protocol, subjects started from a static standing position, and were instructed to perform a descent phase, free knee flexion followed by a rapid and vigorous extension of the lower limb joints (ascent phase). Participants were instructed to jump as high as possible, with trunk as vertically as possible, and with hands remaining on the hips. SJ adopted the same instruction, except that all subjects started the ascent phase with knee flexed (90°) for ~3 s. The jump performance in Team A was performed using an optical sensor system (no trademark), while in Team B, it was measured with an Ergojump contact mat (Cefise, São Paulo, Brazil) processed by the software (Jump fit 1.0, Cefise, São Paulo, Brazil). The dipping phase (CMJ) had a self-selected depth to avoid disturbing the athletes’ coordination. Three attempts were allowed for each jump. Successive attempts of the same jump were made, interspersed by a ~15 s time period. It has been previously reported that optical sensor systems (alternative method) demonstrate strong concurrent validity compared with force platforms (reference method), and excellent test–retest reliability for the estimation of vertical jump height [24].

Five minutes after the jumps, all athletes (Team B) performed three maximal 15 m sprints with a 90 s passive rest interval between each sprint. Each sprint time was recorded by using a photocell system (Cefise, Speed Test 4.0, São Paulo, Brazil), with timing gates placed at the 0 m (i.e., starting gate), 5 m, and 15 m marks (i.e., finishing gates). All sprinting tests were conducted in an indoor court, thus eliminating any potential negative effect of the environmental conditions. The best two jumping and sprinting results were averaged and retained for analysis [25]. In an earlier study, Haugen et al. [26] showed reliability values ranging from 0.7% to 2.8% (coefficient of variation) and from 0.90 to 0.99 (intraclass coefficient of correlation) for the 0–5 m, 0–10 m and 0–20 m sprint times.

### 2.5. Futsal Intermittent Endurance Test (FIET)

After 10 min of the cessation of sprints tests, the athletes (Team B) performed FIET that consisted of shuttle running bouts of 45 m (i.e., 3 × 15 m) performed at progressive speeds until exhaustion was dictated by prerecorded audio cues [27]. Every 45 m, participants are allowed to actively rest for 10 s. After each 8 × 45 m bout, players passively rested for 30 s before continuing. The starting speed was set at 9 km·h^−1^, and speed increments during the first 9 × 45 m bouts were of 0.33 km·h^−1^, successively shifting to 0.20 km·h^−1^ every 45 m, until exhaustion. The test ended when participants did not reach the front line in time, with beeps for two successive times [27]. The peak velocity derived from the FIET (PV_FIET_) was identified as the highest speed that was achieved by the athletes during the test, in km h^−1^. Reproducibility of PV_FIET_ has been previously demonstrated by Castagna et al. [27]. The intraclass correlation coefficient (ICC) and the measurement error, expressed as a coefficient of variation for PV_FIET_ were, respectively, 0.95 and 3.9%.

### 2.6. Session Rating of Perceived Exertion

The ITL was computed using the s-RPE method, as previously used in futsal players [2]. Approximately 15–30 min after the completion of every training session and friendly matches, the players were required to report the intensity of the entire session by the means of a modified 10-point RPE scale [5]. This RPE value was multiplied by the total duration (min) of every training session.

### 2.7. Delayed-Onset Muscle Soreness

Each participant (Team A) was asked to complete a muscle soreness questionnaire for the lower limbs before the warm up of the training session, in which they were required to rank their perception of soreness on a scale from 0 (‘‘absence of soreness’’) to 10 (‘‘very intense soreness’’). This method has been previously used as a non-invasive way to monitor changes in perceived pain, following muscle damaging protocols [28]. Prior to reporting their DOMS ranking, participants were required to perform a standardized half squat with a 90° knee flexion angle, and with the hands fixed on the hips, to ensure that all subjects were experiencing the same movement/sensation [28].

### 2.8. Statistical Analysis

Descriptive statistics are reported as means ± standard deviations (SD). Magnitude based-inference analysis was used to examine the differences in s-RPE TL indicators (including its distribution in different training modes) between both the teams during the preseason period, and to compare the changes in DOMS scores and physical performance outcomes between LTL and HTL groups, using a customized spreadsheet [29,30]. The smallest worthwhile change was calculated (i.e., 0.2 × between-subjects standard deviation SD) and 90% confidence intervals (CI) were also determined. The quantitative chances of higher/beneficial, unclear or lower/harmful differences were evaluated qualitatively as follows: <1%, *almost certainly not*; 1–5%, *very unlikely*; 5–25%, *unlikely*; 25–75%, *possible*; 75–95%, *likely*; 95–99%, *very likely*; >99%, *almost certain*. The true difference was assessed as being unclear when the chances of having positive and negative results were both >5% [29,30]. The standardized mean difference or effect size (ES) was calculated by using the pooled pre-training standard deviation. The criteria to interpret the magnitude of the ES were: ≤0.2 trivial, >0.2–0.6 small, >0.6–1.2 moderate, >1.2–2.0 large, and >2.0–4.0 very large [29]. The Pearson product–moment coefficient of correlation was used to determine if the changes (Δ%) in PV_FIET_ and sprint running performance (5 m and 15 m) were related to each other, with total s-RPE TL, and with their respective baseline values. The threshold used to qualitatively assess the correlations was based on Hopkins et al. [29], using the following criteria: 0.1 trivial; >0.1–0.3 small; >0.3–0.5 moderate; >0.5–0.7 large; >0.7–0.9 very large; >0.9 nearly perfect.

## 3. Results

### 3.1. Comparing s-RPE TL Accumulated during the Preseason between Teams

The percentages of time dedicated to each training purpose throughout the five weeks of the preseason phase for both teams (A and B) are illustrated in Figure 1C. Throughout the preseason phase, the relative time dedicated to technical–tactical, on average, was *likely* (04/07/89) lower in Team B than Team A (41.42 ± 6.44% vs. 49.38 ± 8.19%; ES: −0.78 (90% CI: −1.64–0.08)), while the relative time in aerobic-power/lactate tolerance training was *very likely* (96/02/02) higher in Team B compared to the Team A (18.52 ± 2.82% vs. 7.52 ± 8.59%; ES: 1.03 (90% CI: 0.22–1.83)). The total weekly s-RPE TL (Figure 1A) and training intensity (Figure 1B) differed between both the teams.

### 3.2. Neuromuscular Performance and DOMS in LTL and HTL Groups (Team A)

**Figure 2:**
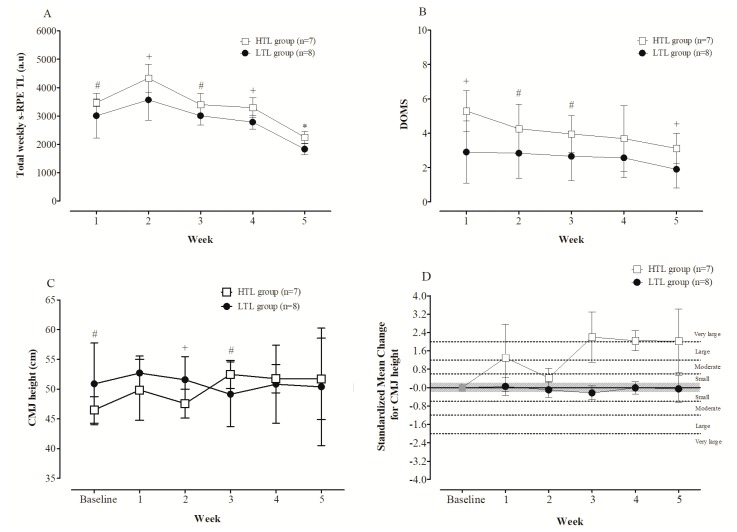
Total weekly s-RPE TL (panel (**A**)), delayed onset of muscle soreness (DOMS) (panel (**B**)), CMJ height (panel (**C**)) and standardized mean changes in CMJ height in relation to the baseline (panel (**D**)) for each group (Low (LTL) and High Training Load (HTL)) over the 5 weeks during the preseason period in professional futsal players. Note: * *almost certain*; + *very likely*; # *likely* differences between groups (HTL vs. LTL).

The total s-RPE TL accumulated during the five weeks of the preseason was 14,184 ± 1638 and 16,732 ± 739 a.u. for LTL (n = 8) and HTL (n = 7) groups, respectively (100% chances of a higher TL for the HTL group, ES = 1.38). Figure 2A displays the total weekly s-RPE TLs over the five week preseason in each group. The total weekly s-RPE TL in week 2 was *very likely* to *almost certainly* higher than those reported in weeks 1, 3, 4, and 5 for both groups.

Figure 2B shows the DOMS values reported throughout the preseason in each group. For the LTL group, changes in the DOMS values between the first four weeks were rated as *unclear*. In week 5, the DOMS value was *likely* lower than those in weeks 2, 3, and 4 (ES ranging from −0.51 (90% CI: −0.95–−0.08) to −0.72 (90% CI: −1.52–0.08)). For HTL group, the DOMS value in week 1 was *likely* to *almost certainly* higher compared to the following weeks (ES ranging from 0.75 (90% CI: −0.06–1.56) to 1.58 (90% CI: 0.87–2.29).

The mean values of CMJ height and standardized mean changes in relation to the baseline across the five weeks of the preseason within each group are presented in Figure 2C,D, respectively. For LTL group, the CMJ height in week 3 was *possibly* lower than at baseline and week 4, and *likely* lower compared to the weeks 1 and 2. All other possible pairwise comparisons were rated as *unclear*. For HTL group, the CMJ height at baseline was *likely* to *almost certainly* lower than the following weeks, while the CMJ height in week 2 was *likely* to *almost certainly* lower, compared to weeks 3, 4, and 5. The standardized mean changes for CMJ height between all weeks in comparison with baseline ranged from small to very large in the HTL group. Otherwise, for the LTL group, with the exception of week 3, which was *possibly* harmful (small effect size), all changes in CMJ height between the remaining weeks in relation to the baseline were rated as trivial/*unclear* (Figure 2D).

The total weekly s-RPE TL were *likely* to *almost certainly* higher in the HTL than in the LTL group across the five weeks (Figure 2A). Except for week 4, which was rated as *unclear* (84/9/7), the DOMS values were *likely* to *very likely* greater in the HTL, compared to the LTL group (Figure 2B). Compared with the LTL group, the HTL group showed *likely* (03/11/86), *unclear* (8/9/84), and *very likely* (01/04/95) lower CMJ heights at baseline, week 1, and week 2, respectively. In week 3, the HTL group had a *likely* (84/13/03) superior CMJ height compared to the LTL group, while no clear and substantial difference between groups in CMJ height was observed for the last two weeks (Figure 2C).

**Figure 3:**
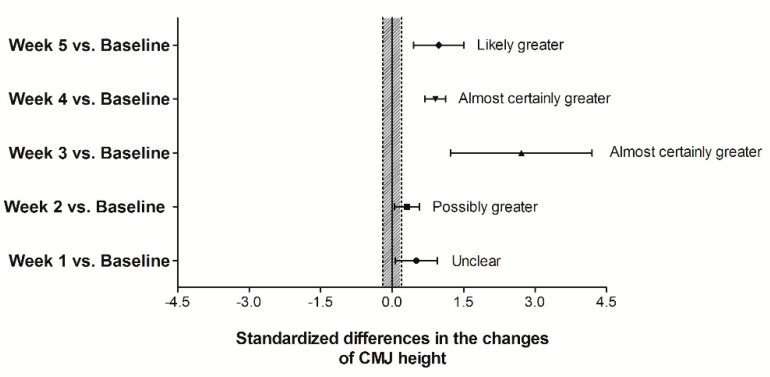
Comparisons (in relation to baseline) of the training-induced changes (Δ) on CMJ height between HTL vs. LTL groups (expressed as the standardized difference). Bars indicate uncertainty in the true mean changes with 90% confidence intervals. The trivial area was calculated from the smallest worthwhile change (see methods). Note: a positive ES denotes a greater change by the HTL group.

The standardized mean differences in the changes (Δ) of CMJ height between LTL and HTL groups are illustrated in Figure 3. When compared to the baseline values, between-groups changes in CMJ height in week 1 was rated as *unclear*, while the magnitude of changes in CMJ height from week 2, 3 and 4, and week 5 were *possibly*, *almost certainly* and *likely* superior in HTL compared to the LTL group, respectively (Figure 3).

### 3.3. Aerobic Performance, Neuromuscular Ability, and Sprinting Speeds and Their Relationships with s-RPE TLs (Team B)

**Figure 4:**
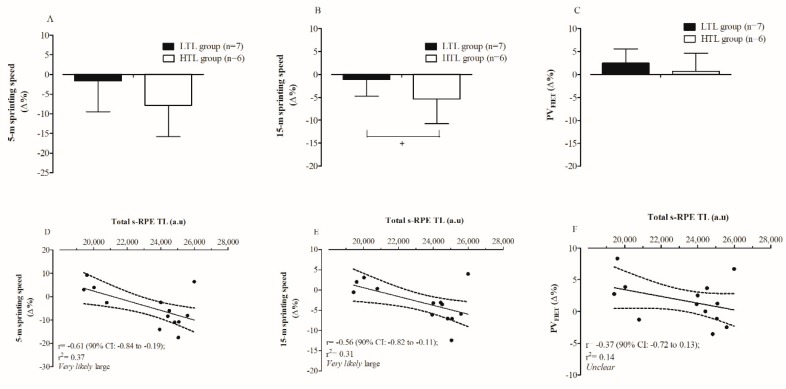
Comparisons between HTL and LTL groups for the changes in 5 m and 15 m sprinting speeds, and PV_FIET_ (panels (**A**–**C**)); and correlation coefficients between total s-RPE TL and changes in 5 m and 15 m sprinting speeds, and PV_FIET_ (panels (**D**–**F**)). Note: + *Likely* difference between groups (High s-RPE vs. Low s-RPE).

The descriptive statistics for all performance parameters, relative changes, and qualitative outcomes resulting from the within-group analysis are presented in Table 1. PV_FIET_ had a *possibly* (59/41/00) small increase from pre- to post-training moment. Changes in CMJ and SJ height were rated as *unclear*. Changes in 5 m and 15 m sprinting speeds were likely harmful after the 5-week preseason phase. Players in the HTL group showed a *likely* greater (95/05/05; ES = −1.14 [90% CI: −2.42–0.14]) reduction in 15 m sprinting speed than LTL group (Figure 4B). In contrast, differences between HTL and LTL groups for the decrement in 5 m sprinting speed were rated as *unclear* (84/10/06; ES = −0.69 (90% CI: −1.57–0.19)) (Figure 4A). No meaningful difference was also found for the changes in PV_FIET_ between HTL and LTL groups (13/16/71; ES: −0.49 (90% CI: −1.50–0.53); Figure 4C). Large negative correlations were found between total s-RPE TL and changes in 5 m and 15 m sprinting speeds (Figure 4D,E).

**Figure 5:**
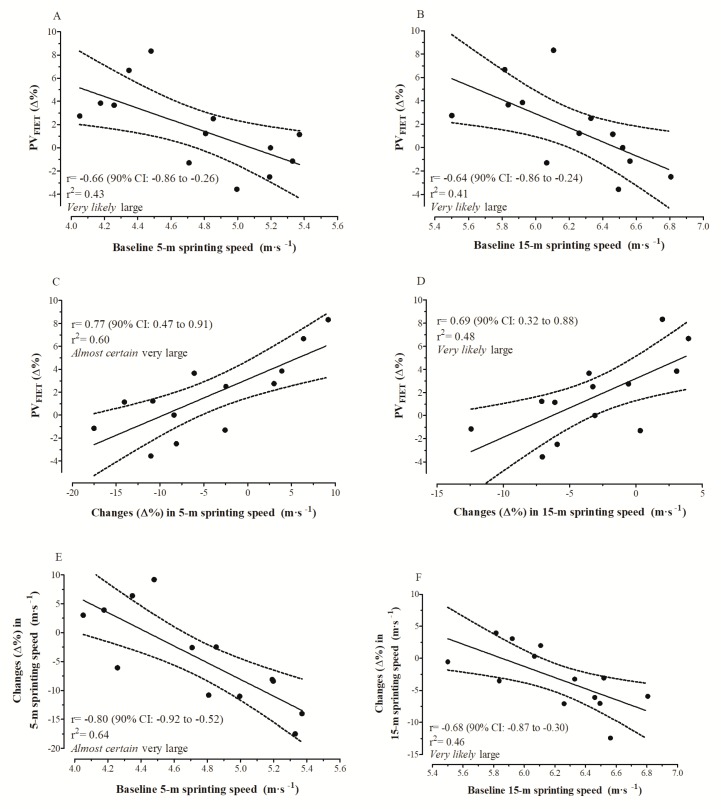
Correlations between changes in PV_FIET_ and baseline 5 m and 15 m sprinting speeds (panels (**A**,**B**)); changes in PV_FIET_ and changes in 5 m and 15 m sprinting speeds (panels (**C**,**D**)); and changes in 5 m and 15 m sprinting speeds, and their respective baseline values (panels (**E**,**F**)).

Figure 5 illustrates the relationship for different combinations between PV_FIET_ and the sprinting speed performance (5 m and 15 m).

## 4. Discussion

The main purposes of the present study were: (1) to describe and compare the typical TL accumulated by two professional Brazilian futsal teams from the First Division League; (2) to compare the weekly changes on neuromuscular performance and DOMS between LTL and HTL groups during the preseason and; (3) to analyze the training-induced changes on aerobic performance, neuromuscular ability, and sprinting speeds in LTL and HTL groups during the preseason, and to explore possible associations with s-RPE TLs and changes in performance.

The major original findings of the current study were as follows: (a) an *almost certainly* to *very likely* difference for weekly s-RPE TL was identified between the two professional futsal teams; (b) the Team A players in HTL group displayed a lower jumping ability at baseline and in the first 2 weeks, as well as reporting a higher DOMS throughout the preseason phase; in addition, players in the HTL group presented superior gains in jumping performance compared to baseline than their peers in LTL group, with large magnitude changes occurring from week 3 onwards, when TLs were reduced; (c) jumping ability and FIET performance showed trivial to small changes, while 5 m and 15 m sprinting speeds were impaired after the five weeks of the preseason phase; such reductions in sprinting speeds were dependent on the athlete’s initial speed characteristics and the total s-RPE TL accumulated during the study period.

### 4.1. Comparing s-RPE TL Accumulated during the Preseason between Teams

Previous studies available have been limited to the monitoring of a single team [2,13,21,22]. The present study adds further information to the literature comparing ITL accumulated during a short preseason period (five weeks) by two different professional teams competing in the major Brazilian Futsal League. The overall relative time devoted to technical–tactical and aerobic-power/lactate tolerance training were different between Team A and Team B (see Figure 1C). This relative training volume distribution is similar to that previously reported by Miloski et al. [2]. However, in spite of similar training strategy distributions, two distinct training loading patterns were identified between clubs. The Team B players accumulated *almost certainly* higher TLs than the Team A players. The magnitudes of the differences were rated as being large to very large. Ferioli et al. [20,31] also found contrasting s-RPE TL between two basketball teams during the preparation period. However, in the study of Ferioli et al. [20,31], the s-RPE TL were compared between professional and semi-professional basketball teams, in opposition to the present study, which compared the s-RPE TL between teams competing in the same National League. This finding has an important practical implication for sport scientists when designing future intervention studies involving different futsal teams, as our data suggest that the s-RPE TL are not similar between the two investigated futsal teams, and this could exert an influence on the main outcomes of the study. Hence, pooling data from different teams in experimental studies (i.e., testing the effectiveness of interventions) should be examined cautiously.

The mean weekly s-RPE TLs accumulated during the preseason phase were 3074 ± 682 a.u. and 4969 ± 544 a.u. for Teams A and B, respectively. These values of ITL in our sample are in agreement with those that are previously reported in several studies in male futsal players [2,13,21,22]. Based on a prior study [2], the mean weekly s-RPE TL in Team A and Team B can be classified as being moderate and high, respectively (Figure 1A). Of relevance, the mean weekly s-RPE TL values accumulated by Team B players are very close to those in response to which the athletes may experience non-functional overreaching/overtraining-related symptoms (i.e., increased severity of upper respiratory tract infection) [9,10].

### 4.2. Neuromuscular Performance and DOMS in LTL and HTL Groups (Team A)

In this study, the HTL group reported higher DOMS throughout the study period, and displayed lower neuromuscular performance than the LTL group at baseline and in the first two weeks of the preseason. It is likely that lower initial lower limb muscle power, as indicated by the difference in baseline CMJ, might have led players from the HTL group to perceive training as being heavier and more fatiguing. Interestingly, it was found that a negative relationship existed between the total s-RPE TL accumulated at week 2 and CMJ height (r = −0.76; p = 0.048) in the HTL group. Further, it should be emphasized that during the highest period of TL (week 2), the players in the HTL group demonstrated the smallest gains (compared to baseline) in jumping ability (Figure 2C). In a study with professional rugby union players, it was also demonstrated that the highest period of training volume (three consecutive weeks) was coincident with a reduced vertical jumping ability in comparison to baseline values (non-fatigued condition), suggesting that neuromuscular fatigue occurred during the heavy phase of preseason [12].

Interestingly, as TLs from week 3 began to reduce, the weekly changes in jumping ability were larger in magnitude compared to the baseline for the HTL group, increasing the CMJ height of this group to similar values to those presented by the players in the LTL group. According to the fitness–fatigue model, it is only when the fatigue-inducing training stimulus has been removed or reduced that improvements in fitness can be observed [32]. Thus, it is suggested that reductions in internal TL can be necessary after intensified training, to allow for gains in neuromuscular performance, especially for those athletes reporting high weekly internal TLs and DOMS scores. The CMJ performance in the LTL group did not improve across the investigated weeks, possibly because players in this group were less trainable (higher initial baseline values). To improve the muscle power associated with jumping action in this group, it is likely that programming more specific training drills (e.g., plyometrics) could have had meaningful results [33].

### 4.3. Aerobic Performance, Neuromuscular Ability, and Sprinting Speeds, and Their Relationships with s-RPE TLs (Team B)

Recent investigations with team sport athletes have demonstrated an inverse relationship between the total s-RPE TL placed upon players, and the changes in power–speed components [19,20]. In this study, the Team B players had their jumping abilities unchanged, and intermittent endurance running performance *possibly* (small ES; 59/41/00%) increased, while sprinting capacities (5 m and 15 m speeds) were impaired in response to the preseason phase. Nakamura et al. [13] also found that the U-20 futsal players experienced substantial decreases in sprinting speeds (5, 10, and 20 m) after a typical futsal preseason (nine weeks). However, the present study is the first showing that reductions in 5 m and 15 m sprinting speeds were largely and negatively correlated with total s-RPE TL accumulated during the five weeks of the preseason period in professional futsal players (Figure 4D,E). A recent observational study with professional and semiprofessional basketball players also found negative relationships between s-RPE TL and weekly training volume, with peripheral neuromuscular function and peak power output evaluated from CMJ [20]. In the referred study of Ferioli et al. [20], the magnitudes of correlations ranged from small to large. Altogether, this information suggests that sustaining high training volumes during the preseason phase might negatively affect strength and power–speed related properties.

Considering the aforementioned evidences, the development of intermittent endurance and power–speed attributes can be positively and adversely affected by high technical–tactical training volumes, respectively. However, it is well recognized that both qualities (i.e., power–speed and aerobic function) are crucial factors for successful performance in futsal [1,34] and therefore they need to be simultaneously optimized. This current investigation found that training-induced changes on intermittent endurance and sprinting capacities were positively related to each other (Figure 5C,D). Prior studies showing this relationship in futsal players are unknown. Our correlation analyses also demonstrated that slower futsal players at the baseline displayed a greater adaptive response than faster ones in improving intermittent endurance and short-term maximal running performances. These preliminary findings highlight that improvements in sprinting speed could be achieved without compromising the intermittent endurance running capacity or vice versa, especially in those futsal players with lower baseline sprinting speeds.

Conversely, faster futsal players can present smaller gains or even reductions in PV_FIET_ because of the larger losses in speed components. This novel finding suggests that impairments in speed-related capacities can negatively influence the magnitude of adaptation in the futsal-specific intermittent endurance running performance of professional athletes. The *very likely* large correlation of 5 m (r = 0.65 (90% CI: 0.25–0.86)) and 15 m (r = 0.56 (90% CI: 0.11–0.82)) sprinting speeds with PV_FIET_ at the beginning of the preseason also confirmed the relevance of the acceleration and speed components during the shuttle run intermittent incremental tests. Thus, coaches need to be advised to take into account the initial speed characteristics of each athlete, to implement individualized and effective training strategies, aiming to ensure that all players have their intermittent endurance running and sprint-related capacities simultaneously improved during the preseason phase. It is important to emphasize in our study the existence of individual players impairing both sprint and intermittent endurance in response to preseason training. The causes of such functional losses and the consequences to the players during the ongoing competition need to be addressed in future studies.

The limitations of the current study are that s-RPE TL was the only TL indicator quantified. No measures of external TL using microtechnology were included, due to their high costs. In addition, the use of different equipment to evaluate the athlete’s vertical jump performance in Team A and B did not allow for training-induced changes in neuromuscular performance to be compared between two teams. To compensate this limitation, changes in power–speed-related capacities were compared within each sample (Team A and B) after dividing players into HTL and LTL groups. This procedure partially enabled an understanding of how distinct TLs schemes could influence the athlete’s adaptive response during the preseason period of elite futsal teams.

## 5. Conclusions

In conclusion, our findings showed that both futsal teams accumulate great TLs during preseason; although higher values were reported in team B. Further, players in the HTL group displayed impairments in jumping performance and high DOMS scores in the first two weeks of the preseason phase. When the TLs were reduced, the HTL group demonstrated better performance in jump height compared to LTL (Team A). For Team B players, sprinting speeds were impaired for the HTL group after five weeks of the preseason phase. In this case, the excessive s-RPE TL accumulated in a short period seems to be among the main reasons for the reduction in the sprint running performance in futsal players of the current sample.

### Practical Applications

As practical application for coaches and technical staffs, our study reinforces the need to monitor TL and DOMS perceptions during the training process, since distinct TL management/distribution and recovery time influences neuromuscular adaptations in futsal players. A descending TL pattern can be an adequate stimulus for maintaining or enhancing vertical jump performance; however, maintenance of a high loading pattern during the entire preseason phase is not recommended, due the impairments caused in sprint running performance. Thus, coaches and trainers are advised to undulate the dynamics of TL applied between microcycles during the preparation period, and to allow recovery cycles. Finally, lower technical–tactical training volume could be planned for the faster players at the end of the week, in order to diminish the overall TL and, in turn, to minimize the negative consequences caused by excessive TL on sprint performance.

## Figures and Tables

**Table 1:** Changes in athletic performance before (pre-training) and after (post-training) the preseason period (five weeks) in elite futsal players (Team B), with relative changes (Δ%), standardized differences (Cohen’s d ± 90% CI) and qualitative outcomes derived from magnitude-based inference analysis.

Performance Measures	Pre-Training (Mean ± sd)	Post-Training (Mean ± sd)	Relative Change (90% CI)	Cohen’s d ± 90% CI	Qualitative Inference (Beneficial/Trivial/Harmful)
PV_FIET_ (km·h^−1^)	15.89 ± 1.00	16.14 ± 0.84	1.55 (−0.10–3.20)	0.23 ± 0.25 (small)	59/41/00 *Possibly*
CMJ (cm)	35.37 ± 3.65	35.75 ± 4.66	1.09 (−4.38–6.55)	0.10 ± 0.50 (trivial)	36/49/15 *Unclear*
SJ (cm)	34.42 ± 4.15	34.56 ± 4.14	0.42 (−3.79–4.64)	0.03 ± 0.33 (trivial)	19/70/11 *Unclear*
5 m sprint (m·s^−1^)	4.75 ± 0.46	4.51 ± 0.26	−10.45 (−19.20–−1.70)	−0.50 ± 0.42 (small)	1/11/88 *Likely*
15 m sprint (m·s^−1^)	6.21 ± 0.37	6.02 ± 0.27	−2.94 (−5.19–−0.69)	−0.46 ± 0.35 (small)	00/11/89 *Likely*

CI: confidence interval; PV_FIET_: Peak velocity derived from Futsal Intermittent Endurance Test (FIET); CMJ: countermovement jump; SJ: Squat Jump.
